# ﻿Caribbean Amphipoda (Crustacea) of Panama. Part III: parvorder Lysianassidira

**DOI:** 10.3897/zookeys.1216.135258

**Published:** 2024-10-24

**Authors:** Kristine N. White

**Affiliations:** 1 Aquatic Sciences Center, Department of Biological and Environmental Sciences, Georgia College & State University, Milledgeville, GA 31061, USA Georgia College & State University Milledgeville United States of America

**Keywords:** Bocas del Toro, identification key, Lysianassidae, Lysianassoidea, Tryphosidae

## Abstract

Amphipods in the parvorder Lysianassidira are scavengers, often collected in sediment, coral rubble, algae, or among other invertebrates. Members of the parvorder have a head that is deeper than long, large coxae, lacinia mobilis present only on the left molar, and a mitten-shaped gnathopod 2 propodus with a long ischium. Nine species from two families within the parvorder are documented from Bocas del Toro, Panama. This research documents range extensions for eight species and an identification key to the species of Caribbean Lysianassidira of Panama is provided.

## Abstract (es)

Los anfípodos del parvorden Lysianassidira son carroñeros, a menudo recolectados en sedimentos, escombros de coral, algas o entre otros invertebrados. Los miembros del parvorden tienen una cabeza que es más profunda que larga, con coxas grandes, lacinia mobilis presenta solo en el molar izquierdo y un gnatópodo 2 en forma de manopla con un isquion largo. Nueve especies de dos familias dentro del parvorder están documentadas en Bocas del Toro, Panamá. Esta investigación documenta extensiones de rango para ocho especies y se proporciona una clave de identificación para las especies de Lysianassidira caribeña de Panamá.

## ﻿Introduction

Parvorder Lysianassidira Dana, 1849 is comprised of 1243 species around the world, with several listed as *incertae sedis* (Horton et al. 2024). Members of the parvorder are characterized by having the head that is deeper than long, antenna 1 with callynophore, large coxae, lacinia mobilis present only on the left molar, and a distally mitten-shaped gnathopod 2 propodus with a long ischium (Lowry and Myers 2017). The parvorder contains 33 families of amphipods: Alicellidae Lowry & DeBroyer, 2008 (17 spp.), Parargissidae Lowry & Myers, 2017 (two spp.), Podoprionidae Lowry & Stoddart, 1996 (four spp.), Valettidae Stebbing, 1888 (two spp.), Valettiopsidae Lowry & DeBroyer, 2008 (12 spp.), Vemanidae Lowry & Myers, 2017 (five spp.), Stegocephalidae Dana, 1852 (110 spp.), Adeliellidae Lowry & Myers, 2017 (three spp.), Amaryllididae Lowry & Stoddart, 2002 (37 spp.), Cebocaridae Lowry & Stoddart, 2011 (15 spp.), Cyclocaridae Lowry & Stoddart, 2011 (four spp.), Cyphocarididae Lowry & Stoddart, 1997 (21 spp.), Eurytheneidae Stoddart & Lowry, 2004 (10 spp.), Hirondelleidae Lowry & Stoddart, 2010a (20 spp.), Lysianassidae Dana, 1849 (130 spp.), Opisidae Lowry & Stoddart, 1995 (19 spp.), Scopelocheiridae Lowry & Stoddart, 1997 (27 spp.), Tryphosidae Lowry & Stoddart, 1997 (389 spp.), Uristidae Hurley, 1963 (190 spp.), Acidostomatidae Stoddart & Lowry, 2012 (11 spp.), Ambasiidae Lowry & Myers, 2017 (three spp.), Aristiidae Lowry & Stoddart, 1997 (42 spp.), Conicostomatidae Lowry & Stoddart, 2012 (19 spp.), Derjugianidae Lowry & Myers, 2017 (one sp.), Endevouridae Lowry & Stoddart, 1997 (19 spp.), Izinkalidae Lowry & Stoddart, 2010c (two spp.), Kergueleniidae Lowry & Stoddart, 2010d (26 spp.), Lepidepecreellidae Stoddart & Lowry, 2010 (12 spp.), Pakynidae Lowry & Myers, 2017 (38 spp.), Sophrosynidae Lowry & Stoddart, 2010b (14 spp.), Thoriellidae Lowry & Stoddart, 2011 (seven spp.), Trischizostomatidae Lilljeborg, 1865 (18 spp.), Wandinidae Lowry & Stoddart, 1990 (four spp.). Only 30 species in the parvorder have been previously reported from the Caribbean Sea, representing ten families (Aristiidae, Cyphocarididae, Endevouridae, Eurythenidae, Lysianassidae, Parargissidae, Stegocephalidae, Tryphosidae, Uristidae, Vemanidae). Four species, *Concarnesconcavus* (Shoemaker, 1933), *Eclecticuseclecticus* Lowry & Stoddart, 1997, *Paracentromedoncarabicus* Barnard, 1964, and *Vemanacompressa* Barnard, 1964 have been previously reported from Caribbean Panama (LeCroy et al. 2009; Miloslavich et al. 2010; Martín et al. 2013). Miloslavich et al. (2010) listed *Parargissagalatheaamericana* Barnard, 1961 from Caribbean Panama without locality details, but Barnard (1961) stated that it was collected from the Pacific. Andres (1977) documented *P.galatheaamericana* from the eastern Atlantic, but the author can find no reports of this species from the Caribbean and, thus, do not include it herein.

Within the parvorder Lysianassidira, nine species of amphipods were collected from Bocas del Toro, Panama, with representatives from the families Lysianassidae and Tryphosidae. Regional diagnoses for each species collected during this study are provided herein. An identification key is provided to distinguish between the Lysianassidira species known from the Caribbean waters of Panama.

## ﻿Methods

Coral rubble, sand, algae, and sponges were collected by hand and placed into buckets or plastic bags from various sites around Bocas del Toro, Panama at depths of 0–15 m. Coral rubble, sand, and algae were elutriated with freshwater to remove amphipods, and sponges were sorted through by hand. Live amphipods were sorted to morphospecies, placed in clove oil for imaging, and preserved in 99.5% EtOH for later examination. Preserved specimens were transferred to glycerol, measured from the tip of the rostrum to the base of the telson, and dissected under a stereomicroscope. Specimens were illustrated using a Meiji MT5900L phase contrast microscope with an Olympus U-DA drawing tube. Illustrations were digitally inked following Coleman (2003) in Adobe Illustrator 2024 using a Wacom^®^ Intuos Pro Pen Tablet. Abbreviations used in figures are as follows: Hd, head; Mx2, maxilla 2; G, gnathopod; P, pereopod; E, epimeron; Ur: urosome; U, uropod; T, telson. Size ranges of each species collected from Bocas del Toro, Panama are provided at the beginning of each material examined section. Specimens are deposited in the Smithsonian Institution, U.S. National Museum of Natural History (**USNM**) and the Gulf Coast Research Laboratory Museum (**GCRL**).

## ﻿Results

**Parvorder Lysianassidira Dana, 1849**

**Superfamily Lysianassoidea Dana, 1849**

**Family Lysianassidae Dana, 1849**

### ﻿Genus Aruga

Taxon classificationAnimaliaAmphipodaLysianassidae

Holmes, 1908

9D0EAF1E-B7F0-5A65-A3A7-13BE75CC28BD

#### Diagnosis.

Antenna 1 with strong callynophore in male and female. Antenna 2 flagellum elongate in male. Epistome not produced; upper lip produced. Maxilla 2 inner plate narrow. Gnathopod 1 simple. Gnathopod 2 minutely chelate. Uropod 2 inner ramus with dorsal notch, gradually narrowing distally. Uropod 3 outer ramus 2-articulate. Telson entire.

### ﻿ Aruga holmesi

Taxon classificationAnimaliaAmphipodaLysianassidae

J.L. Barnard, 1955

6C8AAD1B-8190-566E-88C5-A683B59D4695

Figs 1 , 10A

Aruga holmesi J.L. Barnard, 1955: 100, pls 27, 28; J.L. Barnard 1958: 90; J.L. Barnard 1959: 18; Gurjanova 1962: 299–301, figs 98, 99; J.L. Barnard 1964: 79, chart 1; Barnard and Karaman 1991: 469; Lowry and Stoddart 1997: 47–53, figs 17–20; LeCroy 2007: 575, fig. 497.
Lysianopsis holmesi : Hurley 1963: 74, 75, fig. 21b.
Lysianassa holmesi : J.L. Barnard 1966a: 25; J.L. Barnard 1966b: 69; J.L. Barnard 1979: 12, 130; Austin 1985: 600; Stepien and Brusca 1985: 97–101, fig. 2F; Stretch 1985: 129–133.

#### Material examined.

Panama • 4.8 mm • 1 ♀; Bocas del Toro, Crawl Cay; 9.2376°N, 82.1438°W; depth 1.5–3 m, among coral rubble; 11 Aug 2021; K.N. White leg.; USNM 1739772.

#### Diagnosis.

Upper lip projecting well beyond epistome; epistome concave. Gnathopod 1 propodus posterodistal margin slightly concave. Epimeron 3 posteroventral corner subquadrate, without tooth. Uropod 3 peduncle length at least 2 × width. Telson distal margin truncate, slightly emarginate, with two short setae on each side.

#### Distribution.

USA: Folly Island, South Carolina; Florida from Perdido Key to the lower Florida Keys (LeCroy 2007); Pacific California (Lowry and Stoddart 1997); Ecuador (Lowry and Stoddart 1997); Panama: Pacific side of Isthmus of Panama (Lowry and Stoddart 1997); Bocas del Toro (present study).

**Figure 1.**
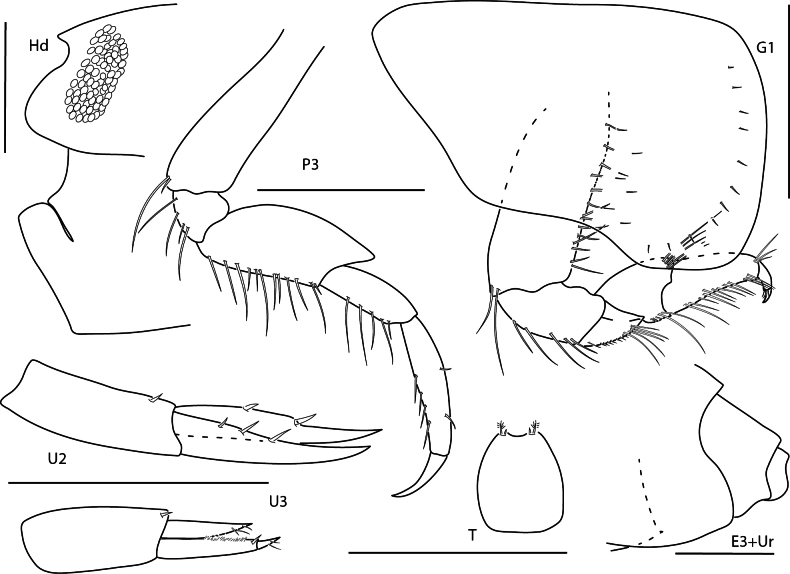
*Arugaholmesi*, female, 4.8 mm, head, epistome and upper lip, pereopod 3, gnathopod 1 lateral, uropod 2, uropod 3, telson, epimeron 3 and urosome. Scale bars: 0.5 mm.

#### Ecology and remarks.

These amphipods are associated with coral rubble and seagrass beds at depths of 1.5–120 m. Panamanian specimens agree closely with previous descriptions of the species. Lowry and Stoddart (1997) recorded this species from the Gulf of Mexico for the first time, noting that it was previously only known from the Pacific side of the Isthmus of Panama. Panamanian specimens are white in color when alive.

### ﻿Genus Bonassa

Taxon classificationAnimaliaAmphipodaLysianassidae

Barnard & Karaman, 1991

7B3E8466-1AF0-5529-8DA4-B003B50327FF

#### Diagnosis.

Antenna 1 with strong callynophore in male. Antenna 2 flagellum elongate in male. Epistome and upper lip produced. Maxilla 2 inner plate narrow. Gnathopod 1 simple. Gnathopod 2 minutely chelate. Uropod 2 inner ramus with dorsal notch, gradually narrowing distally. Uropod 3 outer ramus 1-articulate. Telson entire.

### ﻿ Bonassa bonairensis

Taxon classificationAnimaliaAmphipodaLysianassidae

(Stephensen, 1933)

C69C00FD-769C-5987-8EE7-84C0450CA84D

Figs 2 , 10B

Lysianassa (?) bonairensis Stephensen, 1933a: 416–420, figs 1, 2; Stephensen 1948: 1, 3.
Lysianassa bonairensis J.L. Barnard, 1958: 94; Ortiz 1979: 19.
Bonassa bonairensis Barnard & Karaman, 1991: 472; Lowry and Stoddart 1997: 54–58, figs 21–23.

#### Material examined.

Panama • 2–3 mm • 1 ♀; Bocas del Toro, Swan Cay; 9.4533°N, 82.2983°W; depth 2–3 m, among algae; 4 Aug 2005; S. DeGrave leg.; GCRL 6655 • 1 ♀; Bocas del Toro, Drago; 9.418056°N, 82.3375°W; depth 2–3 m, among coral rubble, 9 Aug 2021; K.N. White leg.; USNM 1739773 • 1 juvenile; Bocas del Toro, Hospital Point; 9.331967°N, 82.214817°W; depth 1–3 m, among coral rubble; 22 June 2023; K.N. White leg.; USNM 1739774.

#### Diagnosis.

Epistome produced, rounded, subequal to produced upper lip. Antenna 1 with strong callynophore in female. Gnathopod 1 propodus distally narrowing. Pereopod 7 basis greatly expanded, posteriorly rounded; merus greatly expanded, approximately 3 × width of carpus. Uropod 3 rami narrow, apically acute, and lacking plumose setae in female. Telson distal margin truncate, slightly emarginate.

**Figure 2.**
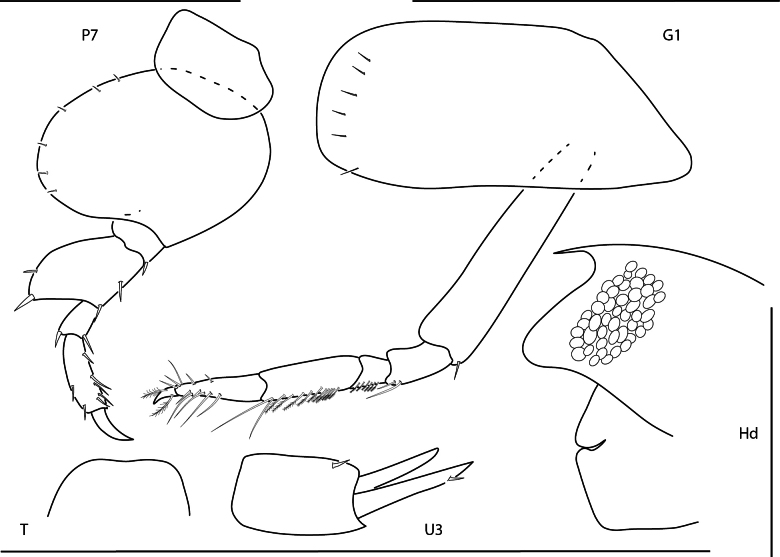
*Bonassabonairensis*, female, 2.8 mm, pereopod 7, gnathopod 1 lateral, telson, uropod 3, head, epistome and upper lip. Scale bars: 0.5 mm.

#### Distribution.

Lesser Antilles: Bonaire Island (Stephensen 1933a; Lowry and Stoddart 1997); Panama: Bocas del Toro (present study).

#### Ecology and remarks.

These amphipods occur among algae and coral rubble at depths of 1–3 m. Panamanian specimens agree closely with previous descriptions of the species, with the exception of a slightly emarginate telson, with the exception of the uropod 3, which is documented for the first time in a female. This species is easily distinguishable based on the expanded pereopod 7 basis and merus. Panamanian specimens are a translucent white color when alive.

### ﻿Genus Concarnes

Taxon classificationAnimaliaAmphipodaLysianassidae

Barnard & Karaman, 1991

8A342C4D-0B62-5301-80A9-845DBAB2D7A1

#### Diagnosis.

Antenna 1 with strong callynophore in male, lacking in female. Antenna 2 flagellum short in male and female. Epistome and upper lip produced. Mouthparts forming quadrate bundle. Maxilla 2 inner plate broad. Gnathopod 1 simple. Uropod 2 inner ramus with dorsal notch, gradually narrowing distally. Uropod 3 outer ramus 2-articulate. Telson weakly cleft.

### ﻿ Concarnes concavus

Taxon classificationAnimaliaAmphipodaLysianassidae

(Shoemaker, 1933)

F321EAD3-A4FE-54FC-A33A-C98D209F26BB

Figs 3 , 10C

Socarnes concavus Shoemaker, 1933: 247–248, fig. 1; J.L. Barnard 1958: 99; Gurjanova 1962: 304; Ortiz 1979: 19.
Concarnes concavus Barnard & Karaman, 1991: 477; Lowry and Stoddart 1997: 58–63, figs 24–26; LeCroy 2007: 576, fig. 493.

#### Material examined.

Panama • 5–6 mm • 1 ♀; Bocas del Toro, Crawl Cay; 9.2475°N, 82.1290°W; depth 5 m, among coral rubble; 12 Aug 2021; K.N. White leg.; USNM 1739775 • 1 ♀; Bocas del Toro, Crawl Cay; 9.2460°N, 82.1369°W; depth 1–4 m, among coral rubble; 25 June 2023; K.N. White leg.; USNM 1739776.

#### Diagnosis.

Head ocular lobe subacute. Epistome produced, rounded, subequal to produced upper lip. Gnathopod 1 basis slender, elongate; propodus distally narrowing. Gnathopod 2 minutely subchelate. Telson partially cleft, lobes apically rounded.

#### Distribution.

USA: Santee River, South Carolina (LeCroy 2007); off Sapelo and Little Tybee Islands, Georgia (LeCroy 2007); Dry Tortugas (Shoemaker 1933); Gulf of Mexico from Florida Keys to Panama City (Thomas 1993; Lowry and Stoddart 1997; LeCroy 2007); Belize (Thomas 1993); Panama: Bocas del Toro (Miloslavich et al. 2010; present study).

**Figure 3.**
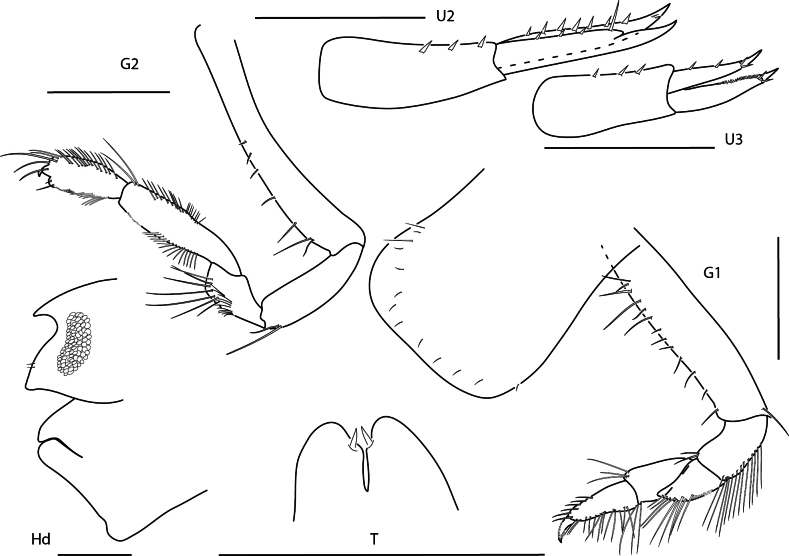
*Concarnesconcavus*, female, 6.0 mm, gnathopod 2 lateral, uropod 2, uropod 3, head, epistome, and upper lip, telson, gnathopod 1 medial. Scale bars: 0.5 mm.

#### Ecology and remarks.

These amphipods are associated with coral rubble and coarse sand at depths of 1–80 m. Panamanian specimens agree closely with previous descriptions of the species. This species is easily recognizable by the subacute ocular lobe, produced epistome and upper lip, and slender, elongate basis of gnathopod 1. Panamanian specimens have a distinct red coloration on the tips of antennae and on the anterior half of the body and have a white snowflake pattern on the posterior half of the body when alive.

### ﻿Genus Lysianopsis

Taxon classificationAnimaliaAmphipodaLysianassidae

Holmes, 1903

FCB28C7F-BF97-549B-A8BF-7F6E77467DC2

#### Diagnosis.

Antenna 1 with strong callynophore in male, weak or lacking in female. Antenna 2 flagellum short in male and female. Epistome not produced; upper lip produced. Maxilla 2 inner plate narrow. Gnathopod 1 simple. Gnathopod 2 minutely chelate. Uropod 2 inner ramus with dorsal notch, gradually narrowing distally. Uropod 3 outer ramus 1-articulate. Telson entire.

### ﻿ Lysianopsis hummelincki

Taxon classificationAnimaliaAmphipodaLysianassidae

(Stephensen, 1933)

D13FA4B9-0A9A-55C2-830A-AEE4DDE79CCB

Figs 4 , 10D

Lysianassa hummelincki Stephensen, 1933b: 438–440, fig. 1; Pirlot 1936: 256; Stephensen 1948: 1, 3, table 1; J.L. Barnard 1958: 94; Hurley 1963: 72; Ortiz 1979: 19.
Lysianassa falcata Stephensen, 1933b: 440–441, fig. 2; Stephensen 1948: 1, 4, table 1; J.L. Barnard 1958: 94; Ortiz 1979: 19.
Lysianopsis alba Barnard & Karaman, 1991: 499 (in part).
Falcanassa falcata Barnard & Karaman, 1991: 486.
Lysianopsis hummelincki Lowry & Stoddart, 1997: 82–89, figs 37–39.

#### Material examined.

Panama • 4 mm • 1 ♂; Bocas del Toro, Hospital Point; 9.3320°N, 82. 2148°W; depth 1–3 m, among coral rubble; 22 June 2023; K.N. White leg.; USNM 1739777.

#### Diagnosis.

Upper lip produced well beyond epistome; epistome straight. Gnathopod 1 of male prehensile. Pereopod 7 basis slightly expanded, posterior margin almost straight, merus slightly expanded, approximately 1.4 × width of carpus. Uropod 3 peduncle length about 1.5 × width; outer ramus 1-articulate. Telson distal margin rounded.

#### Distribution.

Lesser Antilles: Curaçao (Stephensen 1933b); Panama: Bocas del Toro (present study).

#### Ecology and remarks.

These amphipods are associated with sand and coral rubble at depths of intertidal 0–12 m. Panamanian specimens agree closely with previous descriptions with the exception of the almost straight posterior margin on the pereopod 7 basis, which was described by Lowry and Stoddart (1997) as slightly concave. This species is easily recognizable by the 1-articulate outer ramus on uropod 3 and the prehensile gnathopod 1 in males. Panamanian specimens are white with brown spots when alive.

**Figure 4.**
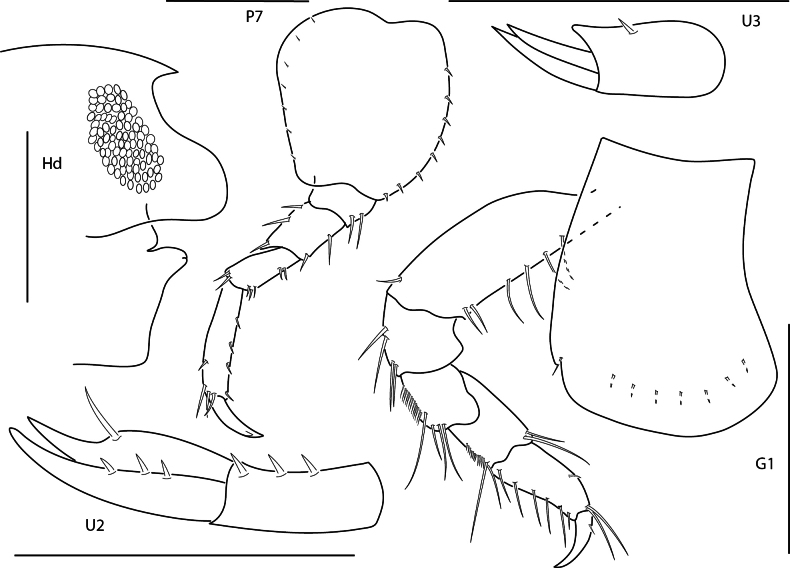
*Lysianopsishummelincki*, male 4.0 mm, head, upper lip, and epistome, pereopod 7, gnathopod 1, uropod 2, uropod 3. Scale bars: 0.5 mm.

### ﻿ Lysianopsis ozona

Taxon classificationAnimaliaAmphipodaLysianassidae

Lowry & Stoddart, 1997

27430BDD-E897-5EF0-8041-A84237D7079E

Figs 5 , 10E

Lysianopsis ozona Lowry & Stoddart, 1997: 87–91, figs 40–42.

#### Material examined.

Panama • 3.2–8.5 mm • 2 ♀; Bocas del Toro, Bastamientos; depth 0–1 m, mangrove scrapings; 1 Aug 2005; T.A. Haney leg.; GCRL 6656. • 2 ♂; Bocas del Toro, Hospital Bight; 9.3045°N, 82.3160°W; depth 1.5 m, among coral rubble; 7 Aug 2005; T.A. Haney leg.; GCRL 6657 • 1 ♀; Bocas del Toro, Marina Bocas; depth 0–1 m, associated with *Phallusianigra* ascidian; 5 June 2009; R. Rocha leg.; GCRL 6658 • 1 ♂; Bocas del Toro, Isla Solarte; 9.2901°N, 82.1897°W; depth 1–5 m, associated with solitary ascidian; 8 Aug 2021; K.N. White leg.; USNM 1739778.

#### Diagnosis.

Epistome concave, subequal to upper lip. Gnathopod 1 propodus posterodistal margin straight; not sexually dimorphic. Uropod 3 peduncle length approximately 1.5 × width; outer ramus 2-articulate. Telson apical margin slightly truncate, apical margin with four short setae medially.

#### Distribution.

USA: Eastern Gulf of Mexico (Lowry and Stoddart 1997); Panama: Bocas del Toro (present study).

#### Ecology and remarks.

These amphipods are associated with sand, coral rubble, and various invertebrates at depths of 0–29 m. Panamanian specimens agree closely with the description provided by Lowry and Stoddart (1997). This species is easily recognizable by the concave epistome and the short uropod 3 peduncle and 2-articulate outer ramus. Panamanian specimens have an orange-brown coloration with white stripes along the pereonite edges when alive.

**Figure 5.**
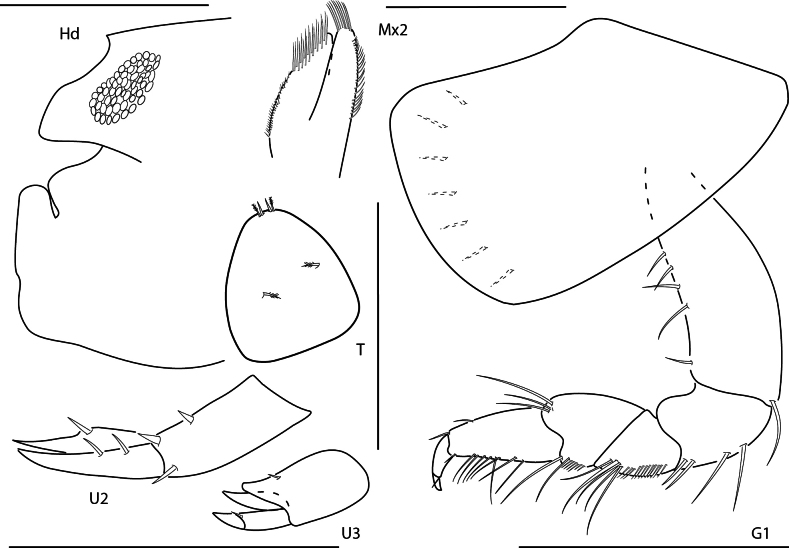
*Lysianopsisozona*, male, 3.2 mm, head, upper lip, and epistome, uropod 2, uropod 3, gnathopod 1 lateral; male, 6.5 mm, maxilla 2, telson. Scale bars: 0.5 mm.

### ﻿Genus Shoemakerella

Taxon classificationAnimaliaAmphipodaLysianassidae

Pirlot, 1936

3A5725C0-F262-5615-947C-E602205B0DD2

#### Diagnosis.

Antenna 1 with weak callynophore in male, lacking in female. Antenna 2 flagellum short in male and female. Epistome not produced; upper lip produced. Maxilla 2 inner plate wider than outer plate. Gnathopod 1 simple. Pereopods 3–4 merus not enlarged compared to carpus. Uropod 2 inner ramus with dorsal notch, abruptly narrowing distally. Uropod 3 outer ramus 1-articulate. Telson entire, dorsal setae inserted proximally (compared to other genera).

### ﻿ Shoemakerella cubensis

Taxon classificationAnimaliaAmphipodaLysianassidae

(Stebbing, 1897)

AFED234F-9004-5EE8-8909-B8B1C2506EB1

Figs 6 , 11A

Lysianax cubensis Stebbing, 1897: 29–30, pl. 7B; Hurley 1963: 70–71, fig. 20 b, c; Lowry and Stoddart 1989: 236–237.
Lysianassa cubensis Stebbing, 1906: 38; Shoemaker 1935: 232–234, fig. 1.
Lysanopsis alba Pearse, 1912: 369, fig. 1 (in part); Shoemaker 1921: 99.
Shoemakerella nasuta Pirlot, 1936: 265–266; Pirlot 1939: 47–48; Shoemaker 1948: 1–2; J.L. Barnard 1969: 180; Ortiz and Lalana Rueda 1993: 26; Ortiz and Lemaitre 1994: 124.
Lysianopsis cubensis Hurley, 1963: fig. 21a.
Lysianassa nasuta Ortiz, 1978: 8; Ortiz 1979: 19; Lalana Rueda and Pérez Moreno 1985: 51; Lalana Rueda et al. 1989: 210; Lalana Rueda and Ortiz 1990: 196; Ortiz and Lalana Rueda 1992: 40.
Shoemakerella cubensis Barnard & Karaman, 1991: 530; Lowry and Stoddart 1997: 92–98, figs 43–45; LeCroy 2007: 588, fig. 495.

#### Material examined.

Panama • 1.5–4 mm • 3 ♀, 1 juvenile; Bocas del Toro, Hospital Point; 9.3336°N, 82.2188°W; depth 15 m, among coral rubble and *Halimeda*; 6 Aug 2005; S. DeGrave and M. Salazar leg.; GCRL 6659 • 2 ♂, 9 ♀, 11 juvenile; Bocas del Toro, Lime Point; 9.4149°N, 82.3323°W; depth 0.2–0.5 m, among coral rubble and red algae; 5 Aug 2005; S. DeGrave and M. Salazar leg.; GCRL 6660 • 1 juvenile; Bocas del Toro, Juan Point; 9.3015°N, 82.2940°W; depth 10 m, among coral rubble; 7 Aug 2021; K.N. White leg.; USNM 1739779 • 1 ♂, 2 juvenile; Bocas del Toro, Isla Solarte; 9.29011°N, 82.1897°W; depth 1–5 m, mangrove scrapings; 8 Aug 2021; K.N. White leg.; USNM 1739780, USNM 1739781.

**Figure 6.**
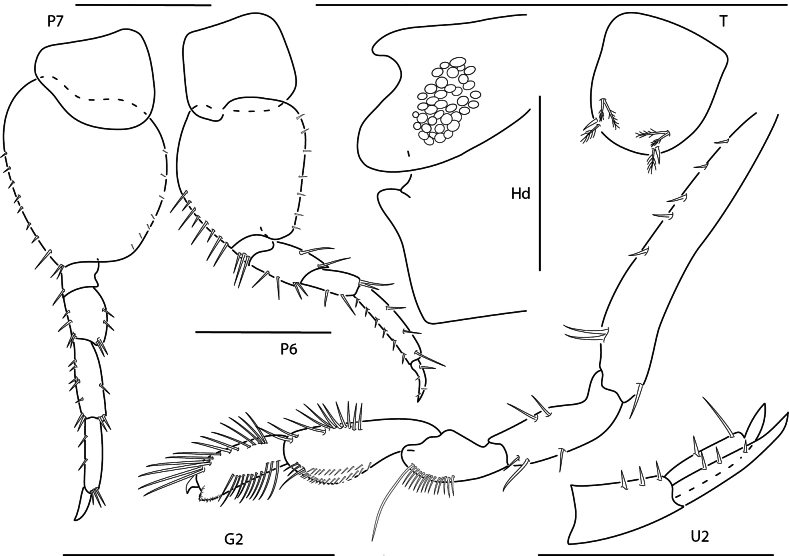
*Shoemakerellacubensis*, male, 4.0 mm, pereopod 7, pereopod 6, head, epistome, and upper lip, telson, gnathopod 2 lateral, uropod 2. Scale bars: 0.5 mm.

#### Diagnosis.

Head and body with tiny setules. Epistome strongly concave. Pereopod 6 basis posterior margin nearly straight. Pereopod 7 propodus length ~5 × width. Telson apex rounded.

#### Distribution.

USA: Panama City to Dry Tortugas, Florida (Lowry and Stoddart 1997; LeCroy 2007); Cuba (Stebbing 1897); Panama: Bocas del Toro (present study).

#### Ecology and remarks.

These amphipods are associated with algae and coral rubble at depths of 2–69 m. Panamanian specimens closely resemble previously described specimens and can be readily distinguished from *Shoemakerellalowryi* Gable & Lazo-Wasem, 1990 based on the pereopod 6 basis posterior margin, pereopod 7 propodus length relative to the carpus length, and the telson apex. Panamanian specimens are yellow-orange in color when alive.

### ﻿ Shoemakerella lowryi

Taxon classificationAnimaliaAmphipodaLysianassidae

Gable & Lazo-Wasem, 1990

E3729DE2-9957-53F4-9ECF-A033CD5DAF0F

Figs 7 , 11B

Lysianassa punctata Kunkel, 1910: 8–10, fig. 1; Johnson 1986: 377, fig. 124.
Shoemakerella lowryi Gable & Lazo-Wasem, 1990: 727–733, figs 5–7.

#### Material examined.

Panama • 2–5.5 mm • 1 ♂; Bocas del Toro, San Cristobal; 9.2625°N, 82.2350°W; depth 15 m, among coral rubble; 10 August 2021; K.N. White leg.; USNM 1739782 • 1 ♀; Bocas del Toro, Swan Cay; 9.4536°N, 82.300033°W; depth 2 m, among sponges; 24 Jun 2023; K.N. White leg.; USNM 1739783 • 2 ♀; Bocas del Toro, Crawl Cay; 9.245967°N, 82.136867°W; depth 1–4 m, among coral rubble; 25 June 2023; K.N. White leg.; USNM 1739784 • 4 ♀; Bocas del Toro, Cayo Zapatilla 1; 9.2700°N, 82.0587°W; depth 10–11 m, among coral rubble; 28 June 2023; K.N. White leg.; USNM 1739785.

#### Diagnosis.

Head and body with tiny setules. Epistome weakly concave. Pereopod 6 basis posterior margin slightly concave. Pereopod 7 propodus length ~9 × width. Telson apex truncate.

**Figure 7.**
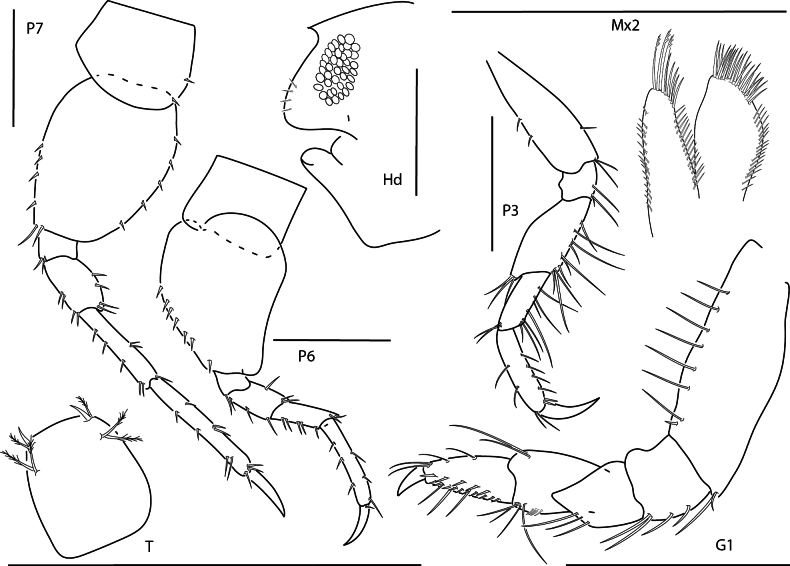
*Shoemakerellalowryi*, male, 4.5 mm, pereopod 7, pereopod 6, head, epistome and upper lip, pereopod 3, maxilla 2, telson, gnathopod 1 lateral. Scale bars: 0.5 mm.

#### Distribution.

Bermuda (Gable and Lazo-Wasem 1990); Panama: Bocas del Toro (present study).

#### Ecology and remarks.

These amphipods are associated with algae, seagrass, and coral rubble at depths of 0.5–9 m. Panamanian specimens closely resemble previously described specimens and can be readily distinguished from *Shoemakerellacubensis* based on the pereopod 6 basis posterior margin, pereopod 7 propodus length relative to the carpus length, and the telson apex. Panamanian specimens are transparent white in color with brown spots when alive.

### ﻿Family Tryphosidae Lowry & Stoddart, 1997

#### ﻿Genus Lepidepecreum

Taxon classificationAnimaliaAmphipodaLysianassidae

Bate & Westwood, 1868

78CF2976-D981-50B8-9320-26EFDD46B837

##### Diagnosis.

Antenna 1 with strong callynophore in male and weak callynophore in female. Antenna 2 of male elongate. Antenna 2 peduncular article 3 elongate in male and female. Maxilla 2 inner plate narrow. Gnathopod 1 subchelate; coxa large, about as long as coxa 2; carpus long (length 2 to 4 × width). Uropod 2 inner ramus without distinct dorsal notch. Uropod 3 outer ramus 2-articulate. Telson cleft.

#### ﻿ Lepidepecreum cf. magdalenensis

Taxon classificationAnimaliaAmphipodaLysianassidae

(Shoemaker, 1942)

32B66617-36BB-5AC2-B3AA-F83D1595E1BB

Figs 8 , 11C

Orchomenella magdalenensis Shoemaker, 1942: 4–7, fig. 1.
Lepidepecreum magdalenensis Lowry & Stoddart, 2002: 173–174; LeCroy 2007: 580, fig. 492.

##### Material examined.

Panama • 2–3 mm • 6 ♂, 16 ♀; Bocas del Toro, Drago Beach; 9.4172°N, 82.3248°W; depth 0–1 m, in sand; 27 June 2023; K.N. White leg.; USNM 1739786.

##### Diagnosis.

Head ocular lobe subrectangular. Gnathopod 1 carpus as long as propodus. Epimeron 3 posteroventral corner subquadrate. Urosomite 1 with dorsodistally acute carina. Uropod 3 inner ramus with two marginal spines.

##### Distribution.

USA: Pacific California (Shoemaker 1942); Florida from Cape Romano to the lower Florida Keys (LeCroy, 2007); Cuba? (Ortiz 1978); Panama: Bocas del Toro (present study).

**Figure 8.**
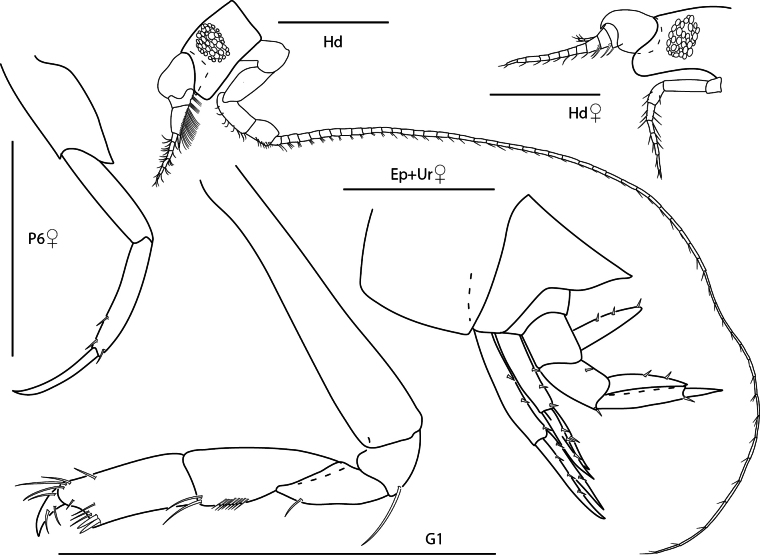
*Lepidepecreummagdalenensis*, female, 3.0 mm, head, epimeron 3 and urosome, pereopod 6; male, 2.8 mm, head, gnathopod 1 lateral. Scale bars: 0.5 mm.

##### Ecology and remarks.

These amphipods are associated with sand at depths of 0.5–27 m. Panamanian specimens closely resemble previously described specimens, except for a weak callynophore in females (strong in original description) and uropod 3 inner ramus having 2 marginal spines (3 in original description). LeCroy (2007) notes that Florida specimens of L.cfmagdalenensis have only one spine, suggesting that this may vary among specimens of this genus. The weak callynophore on antenna 1 of females may suggest that *L.magdalenensis* represents a species complex, but this can only be resolved with further examination of all collections. Panamanian specimens are white in color when alive.

#### ﻿Genus Orchomenella

Taxon classificationAnimaliaAmphipodaLysianassidae

Sars, 1890

0E825C08-58EE-5A37-8EC0-47A44A2C149E

##### Diagnosis.

Antenna 2 of male flagellum elongate. Antenna 2 peduncular article 3 short. Maxilla 2 inner plate narrow. Gnathopod 1 subchelate; carpus short (length less than 2 × width). Uropod 2 inner ramus without distinct dorsal notch. Telson cleft.

#### ﻿ Orchomenella thomasi

Taxon classificationAnimaliaAmphipodaLysianassidae

Lowry & Stoddart, 1997

5477E6BE-8893-5CF9-B18C-DA17F10A7295

Figs 9 , 11D

Orchomenella thomasi Lowry & Stoddart, 1997: 109–113, figs 52–53; LeCroy 2007: 586, fig. 502.

##### Material examined.

Panama • 1.5 mm • 1 ♀; Bocas del Toro, Cayo Zapatilla 1; 9.2700°N, 82.0587°W; depth 10–11 m, among coral rubble; 28 June 2023; K.N. White leg.; USNM 1739787.

##### Diagnosis.

Head ocular lobe subtriangular. Gnathopod 1 carpus shorter than propodus. Epimeron 3 posteroventral corner acute. Urosomite 1 with dorsodistally acute carina. Uropod 3 inner ramus bare; outer ramus 2-articulate.

##### Distribution.

USA: from Sanibel Island, Florida to Louisiana (Lowry and Stoddart 1997; LeCroy 2007); Panama: Bocas del Toro (present study).

**Figure 9.**
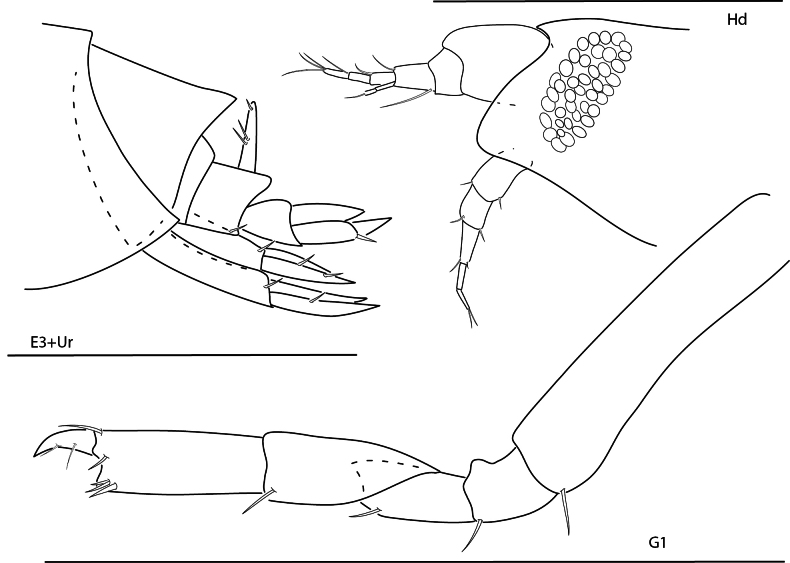
*Orchomenellathomasi*, female, 1.5 mm, head, epimeron 3 and urosome, gnathopod 1 lateral. Scale bars: 0.5 mm.

##### Ecology and remarks.

These amphipods are associated with sand and coral rubble at depths of 10–73 m. Panamanian specimens closely resemble previously described specimens. Panamanian specimens are white in color when alive.

**Figure 10.**
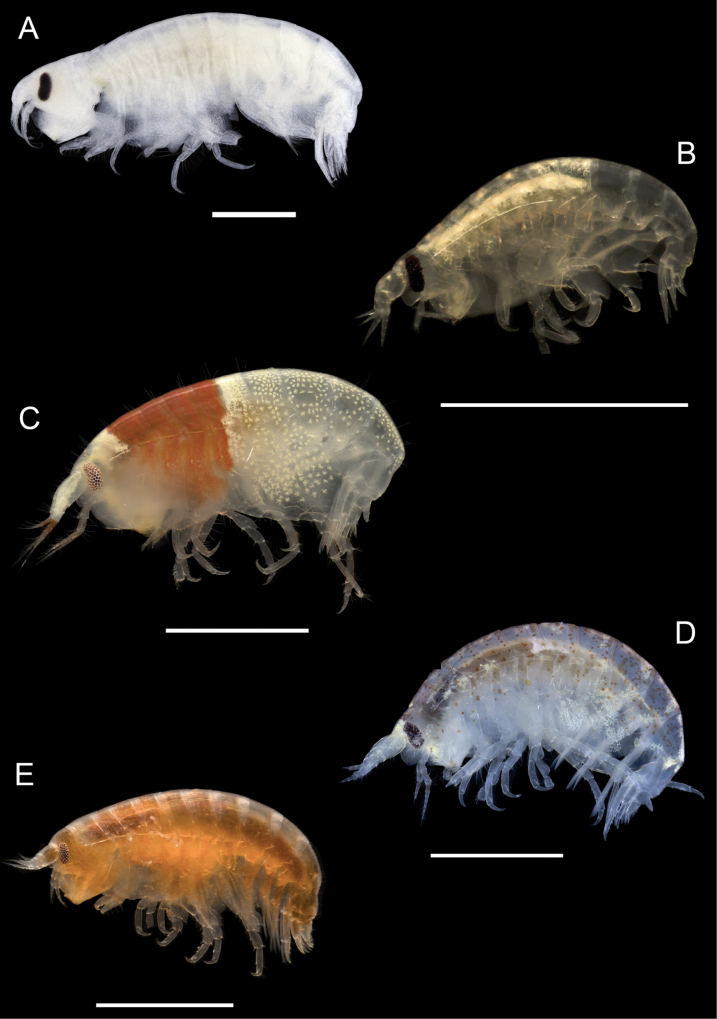
Photographs of live specimens unless noted **A***Arugaholmesi* (ethanol preserved specimen) **B***Bonassabonairensis***C***Concarnesconcavus***D***Lysianopsishummelincki***E***Lysianopsisozona*. Scale bars: 1.0 mm.

**Figure 11.**
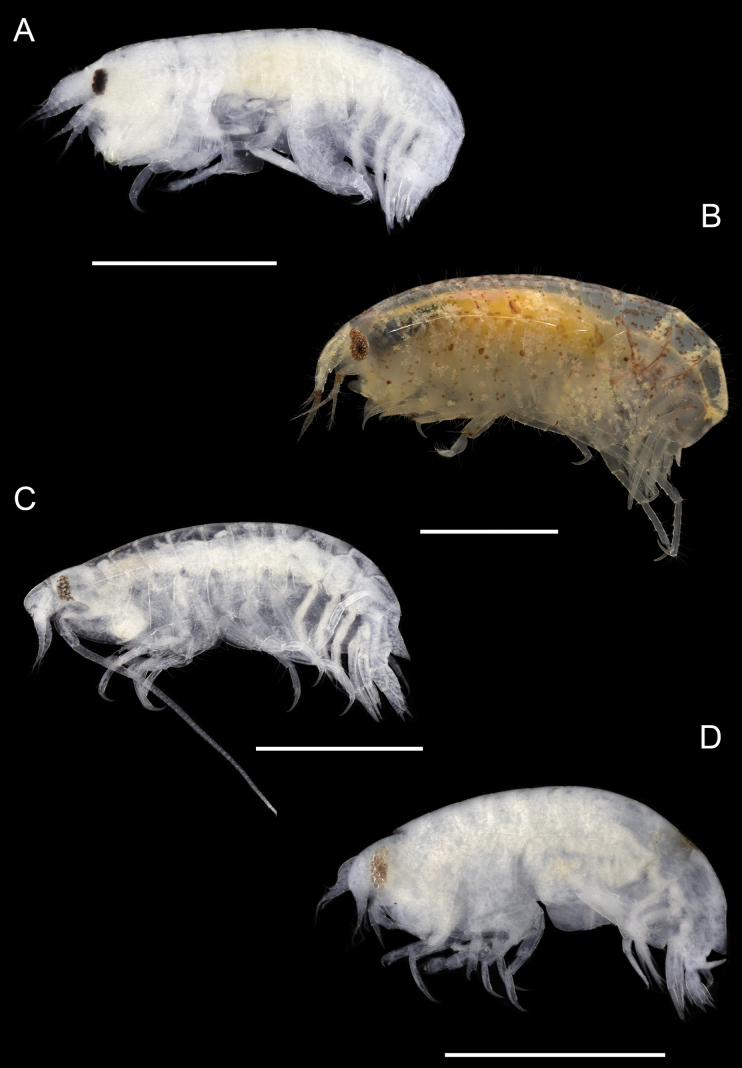
Photographs of live specimens unless noted **A***Shoemakerellacubensis* (ethanol preserved specimen) **B***Shoemakerellalowryi***C***Lepidepecreummagdalenensis* (ethanol preserved specimen) **D***Orchomenellathomasi* (ethanol preserved specimen). Scale bars: 1.0 mm.

#### ﻿Identification Key to the Caribbean Lysianassidira of Panama

**Table:** 

1	Eye absent; pereopod 5 basis narrowly expanded	**2**
–	Eye present, well developed; pereopod 5 basis broadly expanded	**3**
2	Head ocular lobe produced; epimeron 3 posteroventral margin with acute tooth; telson deeply cleft, about 75%	***Paracentromedoncarabicus***
–	Head ocular lobe evenly rounded; epimeron 3 posteroventral margin rounded; telson shallowly cleft, less than 50%	***Vemanacompressa***
3	Gnathopod 1 subchelate; urosomite 1 with dorsodistally acute carina; uropod 2 inner ramus without distinct dorsal notch (Figs 8, 9)	**4**
–	Gnathopod 1 simple; urosomite 1 without dorsodistal carina; uropod 2 inner ramus with distinct dorsal notch (Fig. 1)	**5**
4	Antenna 2 peduncle article 3 long in female; head ocular lobe subrectangular; gnathopod 1 carpus as long as propodus; epimeron 3 posteroventral corner subquadrate; uropod 3 inner ramus with marginal spines (Fig. 8)	***Lepidepecreummagdalenensis***
–	Antenna 2 peduncle article 3 short in female; head ocular lobe subtriangular; gnathopod 1 carpus shorter than propodus; epimeron 3 posteroventral corner acute; uropod 3 inner ramus bare (Fig. 9)	***Orchomenellathomasi***
5	Gnathopod 1 dactylus reduced, complex, covered in long, slender cuticular teeth; telson entire	***Eclecticuseclecticus***
–	Gnathopod 1 dactylus not reduced, simple; telson entire or partially cleft	**6**
6	Gnathopod 2 minutely subchelate; telson partially cleft (Fig. 3)	***Concarnesconcavus***
–	Gnathopod 2 minutely chelate (Figs 5, 6); telson entire (Figs 1, 6)	**7**
7	Maxilla 2 inner plate wider than outer plate (Fig. 7); uropod 2 abruptly narrowing at notch (Fig. 6	**8**
–	Maxilla 2 inner plate narrow, similar in width to outer plate (Fig. 5); uropod 2 gradually narrowing at notch (Fig. 1)	**9**
8	Pereopod 6 basis posterior margin nearly straight; pereopod 7 propodus length ~5 × width; telson apex rounded (Fig. 6)	***Shoemakerellacubensis***
–	Pereopod 6 basis posterior margin slightly concave; pereopod 7 propodus length ~9 × width; telson apex truncate (Fig. 7)	***Shoemakerellalowryi***
9	Epistome rounded; uropod 3 outer ramus 1-articulate (Fig. 2)	**10**
–	Epistome concave; uropod 3 outer ramus 2-articulate (Fig. 1)	**11**
10	Epistome produced, subequal to produced upper lip; gnathopod 1 basis slender; pereopod 7 basis greatly expanded, posteriorly rounded, merus greatly expanded, approximately 3 × width of carpus (Fig. 2)	***Bonassabonairensis***
–	Epistome not produced, upper lip produced; gnathopod 1 basis stout; pereopod 7 basis slightly expanded, posterior margin almost straight, merus slightly expanded, approximately 1.4 × width of carpus (Fig. 4)	***Lysianopsishummelincki***
11	Upper lip projecting well beyond epistome; gnathopod 1 propodus posterodistal margin slightly concave; uropod 3 peduncle long, length at least 2 × width; telson apical margin slightly emarginate (Fig. 1)	***Arugaholmesi***
–	Upper lip subequal to epistome; gnathopod 1 propodus posterodistal margin straight; uropod 3 peduncle short, length approximately 1.5 × width; telson apical margin slightly truncate (Fig. 5)	***Lysianopsisozona***

## ﻿Discussion

The results of this study represent range extensions for eight species of lysianassid amphipods to include the Caribbean waters of Panama. One species collected in this study, *Concarnesconcavus*, has been recorded from the Caribbean of Panama by Miloslavich et al. (2010), yet those authors did not provide any specific locality information, so it is unclear what the exact range of this species is in the Caribbean waters of Panama. Two species documented here have a distribution pattern spanning the eastern Pacific and western Caribbean (*Arugaholmesi* and *Lepidepecreummagdalenensis*). These distribution patterns may suggest that the species were established more than 3 mya, before the isthmus of Panama closed, or that we have species complexes that need to be investigated further.

Characters that have been used to identify lysianassid amphipods in the past, such as setae patterns on the dorsal surface of the body appear to be variable in Panamanian specimens and should not be used for identification. Sexual dimorphism is also used frequently but can be problematic when you have only one specimen or gender. Mouthparts are also often used as diagnostic characters which can be difficult for non-experts; thus, I included as many other characters as possible in this identification key.

## Supplementary Material

XML Treatment for Aruga

XML Treatment for Aruga holmesi

XML Treatment for Bonassa

XML Treatment for Bonassa bonairensis

XML Treatment for Concarnes

XML Treatment for Concarnes concavus

XML Treatment for Lysianopsis

XML Treatment for Lysianopsis hummelincki

XML Treatment for Lysianopsis ozona

XML Treatment for Shoemakerella

XML Treatment for Shoemakerella cubensis

XML Treatment for Shoemakerella lowryi

XML Treatment for Lepidepecreum

XML Treatment for Lepidepecreum cf. magdalenensis

XML Treatment for Orchomenella

XML Treatment for Orchomenella thomasi

## ﻿Additional information

### Author ORCIDs

Kristine N. White https://orcid.org/0000-0002-5203-1656

### Data availability

All of the data that support the findings of this study are available in the main text or Supplementary Information.

## Supplementary materials

Supplementary material 1 Locality table Data type xlsx https://binary.pensoft.net/file/1160288This dataset is made available under the Open Database License (http://opendatacommons.org/licenses/odbl/1.0/). The Open Database License (ODbL) is a license agreement intended to allow users to freely share, modify, and use this Dataset while maintaining this same freedom for others, provided that the original source and author(s) are credited.Author: Kristine N. White
